# Risk factors for infection in patients with a failed kidney allograft on immunosuppressive medications

**DOI:** 10.3389/fneph.2023.1149116

**Published:** 2023-08-21

**Authors:** Lauren Ogawa, Omer E. Beaird, Joanna M. Schaenman

**Affiliations:** Division of Infectious Diseases, David Geffen School of Medicine at University of California—Los Angeles, Los Angeles, CA, United States

**Keywords:** allograft failure, infection, kidney transplantation, immunosuppression, dialysis

## Abstract

Patients with a failing kidney allograft are often continued on immunosuppression (IS) to preserve residual kidney function and prevent allosensitization. It has been previously accepted that maintaining patients on immunosuppressive therapy results in an increased risk of infection, hospitalization, and mortality. However, as the management of IS in patients with a failed kidney allograft continues to evolve, it is important to review the data regarding associations between infection and specific immunosuppression regimens. We present a review of the literature of failed kidney allograft management and infection risk, and discuss practices for infection prevention. Fifteen studies, published from 1995 to 2022, which investigated the experience of patients with failed allograft and infection, were identified. Infection was most commonly documented as a general event, but when specified, included infections caused by *Candida*, *Mycobacterium tuberculosis*, and *Aspergillus.* In addition, the definition of reduced “IS” varied from decreased doses of a triple drug regimen to monotherapy, whereas others did not specify which medications patients were receiving. Despite attempts at lowering net immunosuppression, patients with failed allografts remain at risk of acquiring opportunistic and non-opportunistic infections. Although opportunistic infections secondary to IS are expected, somewhat surprisingly, it appears that the greatest risk of infection may be related to complications of dialysis. Therefore, mitigating strategies, such as planning for an arteriovenous (AV) fistula over a hemodialysis catheter placement, may reduce infection risk. Additional studies are needed to provide more information regarding the types and timing of infection in the setting of a failed kidney allograft. In addition, more data are needed regarding specific medications, doses, and timing of taper of IS to guide future patient management and inform strategies for infection surveillance and prophylaxis.

## 1 Introduction

Patients who have received a kidney transplant are three times more likely to be hospitalized for an infection than for cardiovascular events (1). It is well established that maintenance immunosuppression (IS) in the setting of solid organ transplantation (SOT) carries an increased risk of infection (2). However, less is known about the impact of lower-dose IS in the setting of a failed kidney allograft.

As the number of kidney transplants has increased, a population of patients with failed allografts has emerged, raising the question of how to best manage these patients (3). A previous analysis of US Renal Data System (USRDS) reports indicated that over the past 20 years, 20%–33% of patients experienced graft loss within 5 years of transplantation, with many returning to dialysis (4, 5). Therefore, management of this growing patient population is of concern to both nephrologists and infectious diseases healthcare providers.

There are currently two approaches to the management of patients with a failed kidney allograft: to discontinue IS with or without graft removal or to continue low-dose maintenance IS (6). In addition, the definition of low-dose IS remains unclear and may include discontinuation of drugs or targeting lower drug levels. Therefore, attention to drug class and dosing may be an important aspect for calculation of infectious risk. The continuation of IS has been observed to carry an associated risk of infection (2). As patients with failed allografts often return to dialysis, this may carry additional infectious risks. In this review, we analyze the literature of failed kidney allograft and associated infection risks and discuss practices for infection prevention.

## 2 Methods

A literature search using PubMed^®^ (National Library of Medicine, Bethesda, MD, USA) Advance Search Builder was conducted on 18 December 2022. The search terms (“failed kidney transplant” OR “failed renal transplant” OR “failed kidney allograft” OR “failed renal allograft”) AND (“infection” OR “infectious” OR “bacteria” OR “viral” OR “fungal” OR “parasitic”), excluding “reviews”, “systematic reviews”, and “case reports”, resulted in 454 publications. Our review included prospective, retrospective, observational, and cohort studies in adults and pediatrics. Our search yielded no randomized controlled trials. Of the 454 publications, survey-based studies, and non-kidney transplant- or combination transplant-focused studies were also excluded. Studies that investigated infection-associated mortality or infection complications and risk in the post-failed kidney transplant setting were included in our review. Studies investigating infection as a cause of kidney transplant failure were excluded. A total of 15 studies with full-text availability were included in this review.

## 3 Results

The majority of investigations into infection complications after a failed kidney allograft identified were retrospective studies, with the exception of one multicenter prospective cohort study. The 15 studies included in this review investigated infection risk with a failed kidney transplant, seven of which analyzed for possible associations with IS. Few studies provided information about the specific types of infection and sources of infection.

### 3.1 Immunosuppression and overall infection risk

Multiple studies reported an increase in mortality and infection after kidney allograft failure. A single-center retrospective study, in the Republic of Korea, of 368 patients with a failed allograft found infection to be the second most common cause of death (34.4%) after cardiovascular disease (39.6%) (7). A large retrospective outcome-based study analyzed 78,564 kidney transplants reported to the USRDS from 1988 to 1998 and evaluated the cause of death after allograft loss (8). Over a 10-year follow-up, those patients with a failed allograft had a higher risk of death compared with those with a functional allograft (94.2 *versus* 28.1 per 100 patient-years). Infection was the second most common cause of death after cardiovascular causes and occurred at a higher rate in those with a failed allograft than in those with a functional graft (16.3 *versus* 3.7 per 100 patient-years). The reason for the increased rate of infection in those with allograft failure was unknown, but the authors hypothesized that interventions to save the failing graft could put patients at increased risk of infection. It is also possible that patients were given higher-intensity immunosuppression for the treatment of the rejection (9).

A retrospective study from Canada examined the causes for increased morbidity and mortality in a matched cohort of failing kidney allograft *versus* non-transplant controls with a similar degree of chronic kidney disease (CKD) (10). This study concluded that the patients with a failing allograft had higher rates of hospitalization due to infection (212/520, 40.7%) compared with non-transplant controls with equivalent CKD [71/520, 13.6%, hazard ratio (HR) 3.52; *p*-value < 0.001] and, overall, had higher rates of morbidity and mortality compared with the non-transplant controls. This raises the question of whether or not higher rates of infection resulted from the continued exposure to IS.

Multiple additional studies suggest that the patients who remain on IS in the setting of failed kidney transplantation are at increased risk of infection. A retrospective cohort study from the United States with 186 failed kidney allograft patients found that 44% were hospitalized for fever within the first 6 months after allograft failure (11). Of those hospitalized for fever, patients maintained on full IS had higher rates of infection (15/17, 88%) than those who were weaned off IS (25/65, 38%; *p* < 0.001). However, the overall hospitalization rates were similar in both groups, possibly due to non-infectious fevers related to sensitization and graft intolerance syndrome, which are commonly observed in patients who are weaned off IS (12).

Other retrospective cohort studies have shown similar findings, noting an increased incidence of infection complications in patients with failed kidney allografts maintained on IS (6, 13–15). In two multicenter retrospective cohort studies conducted in the Netherlands, published in 1997 and 2001, Smak Gregooret al. found an increase in mortality from infections associated with IS, even with low-dose maintenance IS, suggesting that there is a benefit to the complete discontinuation of immunosuppressive medications (6, 13). When analyzing infection as a cause for mortality, they found an increase in mortality when comparing patients on low IS with those not on IS (odds ratio (OR) 3.4, *p* < 0.0001). In a more recent 2020 single-center retrospective study of 131 patients with a failed kidney allograft by Ryu et al., it was found that patients who were maintained on IS for 6 months had lower survival rates than receiving low-dose prednisolone (< 10 mg/day) or no steroid (*p* = 0.008) (14). Infection was the second most common cause of death. The investigators recommended the withdrawal of IS and supported the need for appropriate IS weaning protocols.

A Canadian study by Kiberd et al. found infections to be a significant cause of morbidity among failed allograft patients and observed that there was no advantage to tapering IS over a long or short period of time (15). Although the result was not statistically significant, the study found that a longer taper of IS (i.e., > 6 months) had a trend toward higher rates of infection, with a rate of 1.34 infections per year compared with 0.87 infections per year with a shorter taper and 0.72 infections per year in a non-transplant control group. This study was limited by sample size, as only 17 patients were in the long-taper group and 15 in the short-taper group, so it is possible that a larger cohort study may have demonstrated statistical significant differences.

A retrospective study conducted in Canada analyzing the risk factors for sepsis in patients on dialysis after kidney allograft failure found that the rate of sepsis was highest in the first 6 months after allograft failure among the 5,117 patients analyzed (16). Although this study was limited by a lack of information regarding the type of IS, the authors postulated that exposure to immunosuppressive medications increased the risk of sepsis and that tapering IS may decrease that risk. These retrospective studies report on a transplant population with data from the 1970s to 2010s and are limited in that they may not reflect current post-transplant management IS practices.

In the only multicenter prospective study identified, Knoll et al. followed 296 patients with a failed kidney allograft who had recently initiated dialysis (17). Of these 296 patients, 65% were on immunosuppressive medications, such as tacrolimus, cyclosporine, mycophenolate mofetil (MMF), or azathioprine, 20% were on prednisone only, and 15% were off IS. In contrast to the retrospective studies discussed above, Knoll et al. did not find an association between increased risk of death (HR 0.40) or hospitalization (HR 1.81) because of infection and the continued use of IS compared with the discontinuation of all IS or continuation of only prednisone. The majority of infections developed in the first year after graft failure. The investigators emphasized the need for future randomized controlled trials given the contrast in the results and concerns of bias when using observational data.

### 3.2 Opportunistic infections associated with immunosuppression

#### 3.2.1 Known predictors of infection types based on immunosuppression

In SOT, the infection risk is based on patient exposures and the “net state of immunosuppression” (2). One component of the “net state of immunosuppression” is the type, timing, and intensity of immunosuppressive therapy. Therefore, the examination of specific associations with IS types and infection risk may prove useful in selecting a regimen that can maximize benefit without undue infection risk. Understanding the associations between immunosuppressive drug classes and infection can identify the specific infection types that may benefit from prophylaxis or surveillance. For example, the use of calcineurin inhibitors (e.g., tacrolimus, cyclosporine) is associated with human herpes virus [e.g., herpes simplex virus 1 and 2 (HSV-1 and -2), varicella-zoster virus (VZV), and cytomegalovirus (CMV)] infections, gingival infections, and infections due to intracellular pathogens (e.g., mycobacteria and *Toxoplasma gondii*) (2, 18). MMF has been associated with late CMV infection, and a possible increased risk for John Cunningham (JC) virus and progressive multifocal leukoencephalopathy (2, 19). Azathioprine has a possible association with human papillomavirus and increased risk of herpes zoster (2, 20). Corticosteroids are associated with an increased risk for bacterial, fungal [e.g., *Pneumocystis jirovecii (*PJP), aspergillosis, mucormycosis), hepatitis B infection, herpes zoster, and *Strongyloides stercoralis* hyperinfection (2, 21–23).

Due to the enhanced risk for infection, vaccination against preventable diseases is recommended, and the Infectious Diseases Society of America (IDSA) and Advisory Committee on Immunization Practices (ACIP) have published guidelines for the timing and administration of vaccines in the pre- and post-transplant setting (24, 25). Prophylaxis against human herpes viruses, CMV, and fungal and bacterial pathogens is recommended for 3–12 months to a lifetime post transplant depending on the risk and type of transplanted organ (2).

#### 3.2.2 Prevalence of opportunistic infections by type

A retrospective study by Smak Gregoor et al. found that the continuation of IS increases the risk of viral, bacterial, and opportunistic infections (OIs) (6). Viral infections included CMV, VZV, and HSV (OR 7.7, *p* = 0.0002). OIs defined as *Candida*, miliary *Mycobacterium tuberculosis*, *Histoplasma, Aspergillus, Pneumocystis*, and *Cryptococcus* were only seen in those patients remaining on IS (OR 45.3, *p* < 0.0001). Of the 17 OIs reported, 9 out of 17 (52.9%) were due to *Candida*, 3 out of 17 (17.6%) *M. tuberculosis*, 1 out of 17 (5.9%) *Histoplasma*, 1 out of 17 (5.9%) *Aspergillus*, 1 out of 17 (5.9%) *Pneumocystis*, and 2 out of 17 (11.8%) *Cryptococcus*. IS included prednisone, azathioprine, or cyclosporine. However, a limitation of this study was that it remained unclear as to which specific combination of IS or specific monotherapy was associated with infection type.

Woodside et al. analyzed the rates of hospitalization with fever in patients with failed kidney transplants who remained on full IS compared with those on minimal (low-dose prednisone < 10 mg/day) or no IS (11). Full IS was defined as any combination of calcineurin inhibitor, mammalian target of rapamycin (mTOR) inhibitor, MMF, or azathioprine. Of those hospitalized for infection, two from the maintained-on-IS group (2/15, 13.3%) and two from the minimal- or no-IS group (2/25, 8%) were found to have OIs of fungal or mycobacterial etiology. The second most common infection was pyelonephritis, with 3 out of 15 (20%) in the maintained-on-IS group and 4 out of 25 (16%) in the weaned-off-IS or no-IS group. No viral infections were reported. As in the study by Smak Gregoor et al., it remains unclear with which combination of IS therapy these OIs occurred. Although Woodside et al. did find a statistically significant difference in rates of hospitalization with infection amongst the two groups (*p* < 0.001), the association between type of infection and IS regimen was not evaluated.

In a single-center retrospective study comparing the mortality outcomes in failed kidney allografts in patients maintained on or weaned off IS, Ryu et al. reported the incidence of infections, including pneumonia and skin and soft tissue, dialysis-related, gastrointestinal, viral, and urinary tract infections (14). However, infections were reported for the cohort as a whole, rather than by maintained-on *versus* weaned-off IS. Therefore, it is unclear whether specific combinations of immunosuppressive regimens were associated with the type of infection.

#### 3.2.3 Lack of information regarding the association between infection type and regimen

Multiple retrospective studies commented on the variation in management of failed allografts from center to center, immunosuppressive regimens, and the lack of studies (6, 13, 15, 17). For example, some patients are maintained on prednisone or cell cycle inhibitors only, whereas others are on lower doses of all three medications. There is no consensus regarding the definition of low-dose IS or intensity of IS. The majority of studies that have investigated infection complications in the failed kidney allograft setting document infection as a general event rather than describing specific infectious organisms or etiology.

The retrospective study by Kiberd et al. reported that patients received azathioprine, prednisone and a long or short taper of cyclosporine (15). This study concluded that infections were a significant cause of morbidity in both groups regardless of the taper duration but did not define which types of infection and which specific dose or combinations of IS were associated with infection. The supplemental material from the prospective cohort study included average doses of cyclosporine, tacrolimus, MMF, and azathioprine (15). However, there was no discussion as to the specific combinations of immunosuppressive medications or dosages and infection risk.

None of the included studies reported drug levels or patient adherence to immunosuppressive medications, making it challenging to conclude which regimens and doses of IS are associated with an increased risk of infection.

### 3.3 Allograft failure and dialysis as a source of non-opportunistic infection

Another aspect in the management of patients with a failed kidney allograft is the decision to reinitiate dialysis. Those with a failed kidney allograft often remain on immunosuppressive medications to maintain residual kidney allograft function (9). However, kidney function can continue to decline, thus requiring initiation of peritoneal (PD) or hemodialysis (HD) and adding another risk for infection complications. Dialysis is not benign, and infection is the second leading cause of mortality among dialysis patients, accounting for 8% of all deaths (26). Dialysis-dependent patients are at an increased risk of infection from contaminated equipment, frequent manipulation of catheters, and surgical site complications (27). The infection risk associated with dialysis in the failed kidney allograft setting has been analyzed in several studies.

In a 2007 Canadian retrospective study, Gill et al. analyzed mortality rates in patients on the waitlist for renal replacement therapy, those with a functioning allograft, and those with allograft failure who were returning to dialysis (28). Among the three groups, those with allograft failure transitioning to dialysis had the highest rate of death (17.9/100 patient-years), which peaked at 3 months after allograft failure. There was a higher incidence of sepsis in the failed allograft group (16.8%) than in the waitlist (14%) and functional allograft groups (12.7%), and those receiving HD had higher mortality rates (10.7/100 patient-years) than those on PD (7.9/100 patient-years). The role of IS and cause of sepsis while on dialysis were not evaluated, so it is unknown if sepsis was related to catheters, peritonitis, or other etiologies. The investigators suggested that weaning patients off IS and creating arteriovenous vascular access may decrease mortality.

Another study of failed kidney transplant patients who were hospitalized for fever in the first 6 months post-allograft failure concluded that the patients maintained on full IS were at greater risk of infection than those weaned off IS or maintained on low-dose regimens (11). Of those with an infection, from the maintained-on-IS group, 5 out of 11 (33%) cases were HD line-associated infections, compared with 11 out of 65 (16.9%) in the weaned-off-IS group. HD line-associated infections were the most common cause of infection, regardless of whether or not patients had been weaned off immunosuppression.

When comparing a short IS taper, long IS taper, and a non-transplanted control group, two-thirds (45/67, 67.2%) of infections were dialysis related (15). All three groups had similar types of dialysis-related infections, including an infected fistula (5/45, 11.1%) and HD catheter (8/45, 17.8%), peritonitis (23/45, 51.1%), and a peritoneal exit site infection (9/45, 20%). Again, regardless of IS, the infection risk was largely associated with dialysis.

A retrospective study focusing on failed kidney allograft patients and the incidence of sepsis found that those treated with HD after failure had an increased risk of sepsis (HR 1.70, *p* < 0.001) (16). In this study, the investigators questioned whether or not the type of vascular access contributed to the high rate of sepsis during the first 6 months after transplant failure. This question was further addressed in a retrospective study from Argentina of 138 patients with a failed kidney allograft who were on dialysis (29). Eighty-five (61.6%) patients were categorized into a programmed vascular access group, who had received an AV fistula or polytetrafluorethylene graft. This group was compared with 53 (38.4%) patients who had unprogrammed vascular access, defined as a tunneled or non-tunneled catheter. This study observed a difference in the rate of mortality in the unprogrammed vascular access group (22/53, 41.5%) compared with the programmed vascular access group (7/85, 8.2%) with a failed kidney graft. There was a statistically significant association between having a HD catheter and increased mortality (HR 5.904, *p* < 0.0001). Infection was the second most frequent (27.3%) cause of mortality, although the type of infection was not specified. Therefore, it is unclear if mortality was related to a line infection or other infection etiologies.

Chaudhri et al. reported on a small single-center retrospective study of peritoneal dialysis outcomes in 50 patients with a failed kidney transplant (30). Patients were compared with non-transplant patients on PD. Thirty-eight of the failed allograft patients reported information regarding IS, which was reduced corticosteroids with or without a calcineurin inhibitor. There was no significant difference in patient survival between the two groups, and rates of peritonitis were similar (1 episode per 29.3 months *versus* 1 episode per 22.8 months in the control group). This study was limited by sample size, but a larger retrospective study conducted in France had similar findings.

The French study by Benomar et al., comparing 358 patients with a failed kidney transplant on PD to 656 non-transplanted patients on PD, also found no statistically significant differences in survival (*p* = 0.34) or rates of peritonitis (*p* = 0.3) (31). Although no data on specific IS was collected in this study, the results suggest that IS may not have an association with increased rates of peritonitis as there was no difference found between the failed kidney transplant and non-transplant groups. This report supports the hypothesis that PD may be a safer option than HD for patients on reduced IS awaiting re-transplantation.

A single-center retrospective study from the Republic of Korea compared three patient groups: those with a failed kidney allograft on HD, PD, or who had undergone re-transplantation (7). All subjects were on cyclosporine or tacrolimus and prednisone, with medications discontinued 3–6 months after allograft failure. Infection was the second most common cause of death in all three groups, and there were no significant differences in the distribution of infection (*p* = 0.54) among the groups. The etiology of the infection was not reported, so it is unclear whether infections were related to complications from dialysis or associated with IS. This further highlights the need for additional studies comparing the various renal replacement modalities, infection risk, and etiologies of infection.

No association between IS and the incidence of peritonitis was found in a retrospective pediatric study comparing failed kidney allograft on PD to transplant-naive patients on PD (32). The failed allograft group had higher rates of peritonitis, although this was not statistically significant compared with the transplant-naive group (*p* = 0.56). The population of this study was patients on PD, and it is unclear how rates of peritonitis in this population would compare with those in pediatric patients on other renal replacement therapies. The investigators highlighted that there is limited to no information in the pediatric literature comparing IS and dialysis morbidity and mortality or guidance for the management of pediatric patients with a failed allograft.

These studies all highlight the need for additional studies comparing the various renal replacement modalities, infection risk, and etiologies of infection.

### 3.4 Additional risk factors for infection

The continued use of IS and dialysis are not the only factors that have been suggested to increase the risk of infection with a failed kidney transplant. The retrospective Canadian study conducted by Johnston et al. investigated risk factors and consequences of septicemia in patients on dialysis after kidney allograft failure (16). In addition to HD, the study found that patients over the age of 60 years and with diabetes were at a statistically significant higher risk of septicemia (*p* < 0.001). Other risk factors for sepsis after allograft failure included obesity, history of peripheral vascular disease, and congestive heart failure. Further evaluation at the genetic or molecular level was not conducted.

A pediatric study of compared infection risk when on PD among transplant-naive patients to those with a failed kidney allograft (32). Within a cohort of 2,829 patients, those who were black were at a higher risk of peritonitis and 44% more likely to develop peritonitis than those who were white (*p* < 0.001). This finding is supported by several other studies in both pediatric and adult populations; however, it is unclear if this applies to transplant failure alone (33, 34). The investigators suggest the need for additional studies to evaluate the association between race-, infection-, and dialysis-related mortality.

## 4 Discussion

**Figure 1:**
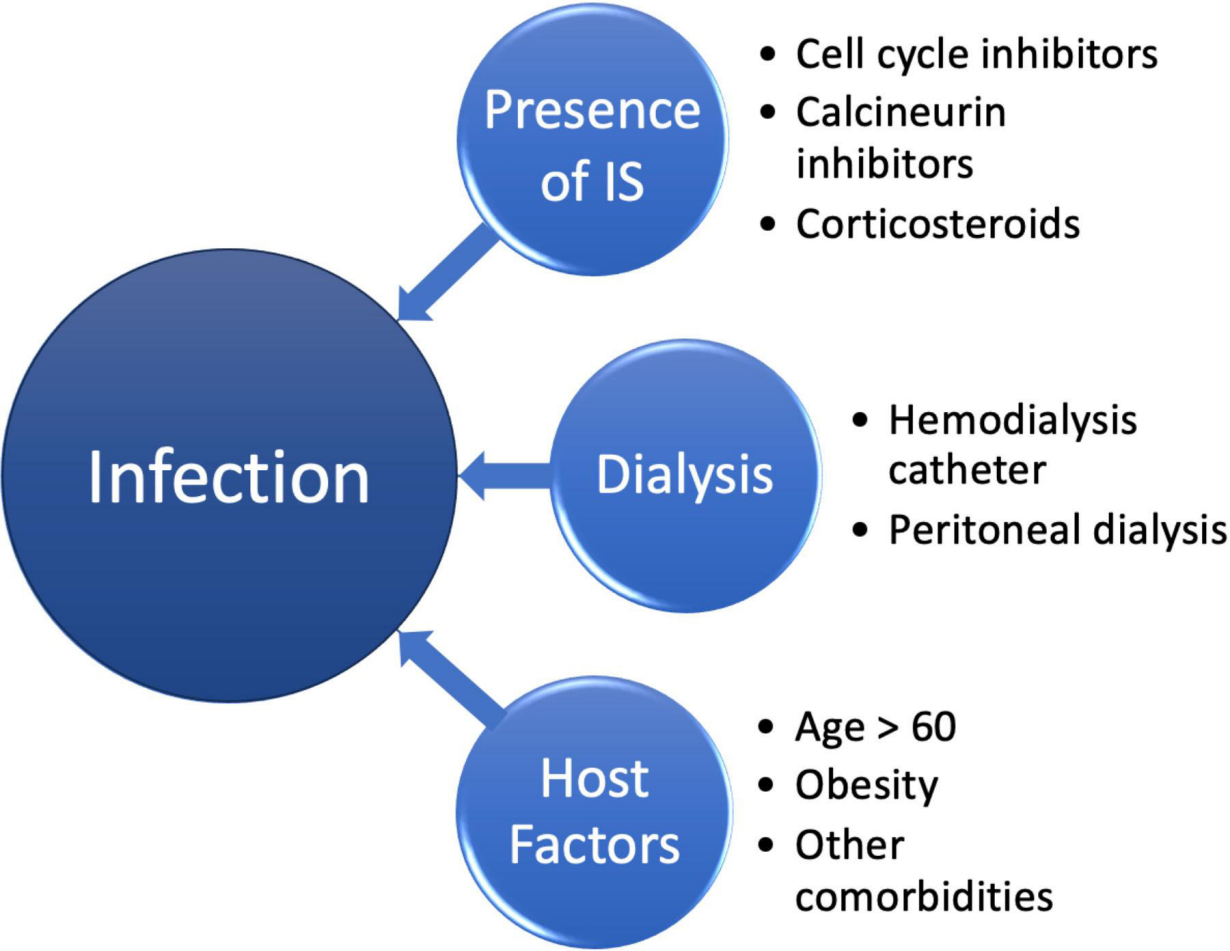
Risk factors for infection in the setting of failed allograft.

It is widely accepted that immunosuppressive medications increase the risk of infection. Risk is often determined by the exposure and intensity of IS. This risk persists in patients who have a failed kidney allograft, as demonstrated by the retrospective studies included in this review. In addition to IS, other risk factors for infection in the setting of a failed allograft include dialysis, older age, and comorbidities (**Figure 1**).

Due to the risk of infection complications from IS, the discontinuation of IS in this setting has been recommended (6, 13). However, IS is typically continued to preserve residual kidney function and prevent allosensitization as stopping IS could impact patients’ candidacy for re-transplantation (9, 35). These retrospective studies do not include recent advancements in failed allograft management and may not reflect current practices. Pham et al. and Davis et al. have both proposed methods of tapering IS, and additional studies would aid in determining infection risk when following a more current immunosuppressive medication regimen (4, 35).

Concerns for infection when remaining on IS after allograft failure have been challenged by the 2022 prospective cohort study from Canada, which, in contrast, showed that the continuation of immunosuppressive medications was associated with a lower risk of death (17). This study also demonstrated that IS was not associated with an increased risk of hospitalization due to infection. Due to these differences in findings and recent changes in the management of allograft failure, additional studies are warranted.

Previous studies have been limited by a lack of information regarding drug levels and dosing of IS and the heterogeneity of regimens associated with type of infections. It is therefore challenging to draw conclusions on the impact of infection risk between lower-dose triple-drug IS therapy, prednisone alone, or other monotherapies. The implication of regimen type and drug levels on infection risk is an area of potential further study. There are limited to no data for prophylaxis use in the setting of kidney allograft failure. Suggestions for assessing the level of infection risk and associated immunosuppressants and methods to reduce risk are presented in **Table 1**.

**Table 1:** Failed renal allograft on ‘low dose’ immunosuppression– strategies to minimize infection risk.

	Risk level	Associated IS (2, 36)	Surveillance	Prophylaxis^**^
PJP/PCP (37, 38)	+	All	Monitor for evidence of pneumonia symptoms	None, if <20mg/day of prednisone (or equivalent )
*Aspergillus* (39)	+	All	Monitor for evidence of pneumonia symptoms	None
Endemic fungi (40)	+	All	Screening serology for *Coccidioides* ^*^	Azole if living in *Coccidioides* endemic area or history of infection if on IS
CMV (41, 42)	++	CNI or cell cycle inhibitors	Serum PCR if concern for disease	None
HSV/VZV (43)		All	Monitor for evidence of skin infection	Consider acyclovir if history of severe or recurrent disease; vaccinate with recombinant shingles vaccine
MTB (44)	+	All	MTB interferon release assay if not performed previously	Latent TB Rx if not performed previously
*Candida* (45)	++	Corticosteroids	Monitor for evidence of abdominal, mucosal, skin infection	None
Line infection (24)	+++	N/A	Monitor for evidence of systemic symptoms including fever	None

IS, immunosuppression; PJP/PCP, Pneumocystis jiroveci (carinii); TMP-SMX, Trimethoprim-sulfamethoxazole; CNI, Calcineurin inhibitor; CMV, Cytomegalovirus; HSV, Herpes simplex virus; VZV, Varicella zoster virus; MTB, Mycobacterium tuberculosis; N/A, not applicable.

+, low risk; ++, moderate risk; +++, high risk.

* Screening serology with *Coccidioides* IgG EIA or if unavailable *Coccidioides* ID and CF Ab. Screening for *Histoplasma* and *Blastomyces* not routinely recommended. Risk for *Coccidioides* include previous or current residence in the Southwest or Mexico.

**If recently transplanted (and acutely failed) or treated for rejection would continue standard prophylaxis for at least 3 months for PJP, endemic fungi, and viral (HSV/CMV) before transitioning to recommendations included in this table-Provided references are for general information about prophylaxis and associated immunosuppression.  Recommendations for degree of risk, screening post-failed allograft, and indications for prophylaxis are based on the authors’ expert opinion.

In the majority of studies, infection was documented as a general event. Few studies provided data regarding infection type (e.g., fungal, bacterial, or viral). In one of the two studies that did provide more specific data on infection etiology, Woodside et al. found that of those patients who were weaned off IS, 8% had an OI in the first 6 months after failure (11). In contrast, the incidence of specific infections has been studied in kidney transplantation, with the greatest risk of infection occurring, on average, 3 months post transplantation, when patients are often on the highest doses of immunosuppressants (2). Typically, the intensity of IS for the majority of patients decreases after 6 months post-transplant, and patients are more commonly at risk for community-acquired infection or infection associated with environmental exposures (2, 46). Since patients with a failed kidney allograft are also on reduced-intensity IS, their infection risk is likely to be slightly lower than patients with a functional graft 6 months post-transplant on stable IS. Therefore, they are at greater risk for community-acquired or environmental-associated infections rather than OIs. However, if reinitiated on dialysis, there is additional risk for dialysis-associated infections.

One review of infection outcomes post-kidney transplantation found the incidence of CMV infection to be 8%–32%, HSV infection to be 53%, VZV infection to be 4%–12%, and bacterial infection to be 47% (46). A cohort study evaluating the outcomes of OIs in 538 patients after kidney transplantation in France, who were followed for a mean of 55 months, found that 15% had an OI and that 48% of those infections occurred within the first year post-transplantation (47). Of the whole cohort, 10% experienced a viral OI, 3% a fungal OI, and 1% a parasitic infection. Comparing an 8% incidence of OIs in failed kidney allograft patients from Woodside et al. with the incidences reported among patients with functional allografts demonstrates that there is a lower incidence of OIs in those with a failed graft. This raises the possibility that reduced IS is protective in decreasing the risk of infection. More studies are required to validate this possible conclusion.

A review of the literature did not yield data on the prophylaxis or surveillance for patients remaining on IS with a failed kidney allograft. In the studies reviewed, it is unknown if infection occurred in the presence or absence of prophylaxis. Given the variability in IS regimens, future studies are warranted to investigate the role of prophylaxis *versus* pre-emptive monitoring in the setting of failed kidney transplantation.

Retrospective studies have suggested that, rather than OIs related to continued IS, the greatest risk may be due to complications from dialysis, such as catheter-related infections. There was greater mortality in patients who returned to HD with a catheter, and HD line-associated infection was the most common infection regardless of IS (11, 29). Therefore, efforts to prevent infection should continue after allograft failure. Infection is the second highest cause of mortality among those on dialysis (27). The Centers for Disease Control and Prevention (CDC), Healthcare Infection Control Practices Advisory Committee, and National Kidney Foundation have developed guidelines to prevent bloodstream infections in dialysis patients, which include interventions focusing on hand hygiene, patient and clinical staff education, and catheter manipulation and sterilization techniques (48, 49). More advanced planning when transitioning to dialysis and arranging for an AV fistula or graft instead of a catheter may be beneficial to reduce the mortality risk and infection complications.

An alternative is to utilize PD, thus avoiding an invasive venous catheter. Three studies in this review investigated outcomes in patients with failed kidney allografts on PD compared with non-transplant patients and found no differences in the rates of infection or peritonitis (30–32). No association was seen between infection or peritonitis and the use of IS. These studies had either a small sample size or were single center, and so it is unclear if the results are generalizable to larger populations. Larger prospective or controlled trials could aid in comparing various renal replacement therapies, IS regimens, and types of infection.

Another component of infection prevention is staying up to date with vaccinations as kidney transplant recipients are at an increased risk of vaccine-preventable diseases compared with patients with CKD alone. It is recommended that vaccination schedules are resumed 3–6 months after kidney transplantation (50). Following ACIP guidelines, providers should ensure that patients are vaccinated against hepatitis A, hepatitis B, pneumococcus, influenza, human papillomavirus, tetanus, pertussis, diphtheria, meningococcus, herpes zoster, and COVID-19. In patients with a failed kidney allograft who remain on IS, live vaccines should be avoided.

In conclusion, the management of patients with a failed or failing kidney allograft may benefit from a multidisciplinary approach given the risks for infection- and non-infection-related complications. A review of retrospective studies indicated that those with a failed kidney allograft who are continued on immunosuppression are at risk of infection; however, the greatest infection-related concern may be due to complications from dialysis, such as HD catheter infections. Due to the dynamic and evolving field of transplant medicine, future studies are needed, along with continued collaboration between nephrology and infectious disease healthcare providers to improve patient care and outcomes.

## Author contributions

LO and JS contributed to conception and design of this review. LO performed the literature review and wrote the manuscript. OB and JS wrote the manuscript and contributed critical evaluation. All authors contributed to table design and content, manuscript revision, and approved the submitted final version.
